# Demonstration of laser cooling in a novel all oxide GAYY silica glass

**DOI:** 10.1038/s41598-023-31912-1

**Published:** 2023-04-03

**Authors:** Jyothis Thomas, Thomas Meyneng, Amirhossein Tehranchi, Nicolas Gregoire, Frederic Monet, Denis Seletskiy, Younès Messaddeq, Raman Kashyap

**Affiliations:** 1 Fabulas Laboratory, Department of Engineering Physics, Polytechnique Montreal, 2900 Blvd Edouard-Montpetit, Montreal, H3T 1J4 Canada; 2 Centre d’Optique, Photonique et Laser, Université Laval, 2375 Rue de la Terrasse, Québec, QC G1V 0A6 Canada; 3 Fabulas Laboratory, Department of Electrical Engineering, Polytechnique Montreal, 2900 Blvd Edouard-Montpetit, Montreal, H3T 1J4 Canada; 4 femtoQ Laboratory, Department of Engineering Physics, Polytechnique Montreal, 2900 Blvd Edouard-Montpetit, Montreal, H3T 1J4 Canada

**Keywords:** Optical materials and structures, Materials for optics

## Abstract

We demonstrate laser induced cooling in ytterbium doped silica (SiO_2_) glass with alumina, yttria co-doping (GAYY-Aluminum: Yttrium: Ytterbium Glass) fabricated using the modified chemical vapour deposition (MCVD) technique. A maximum temperature reduction by − 0.9 K from room temperature (296 K) at atmospheric pressure was achieved using only 6.5 W of 1029 nm laser radiation. The developed fabrication process allows us to incorporate ytterbium at concentration of 4 × 10^26^ ions/m^3^ which is the highest value reported for laser cooling without clustering or lifetime shortening, as well as to reach a very low background absorptive loss of 10 dB/km. The numerical simulation of temperature change versus pump power well agrees with the observation and predicts, for the same conditions, a temperature reduction of 4 K from room temperature in a vacuum. This novel silica glass has a high potential for a vast number of applications in laser cooling such as radiation-balanced amplifiers and high-power lasers including fiber lasers.

## Introduction

Optical cooling of solids was first postulated by Pringsheim in 1929^1^ via anti-Stokes emission of radiation. It took 65 years for the stringent requirements of material properties to be met before it could be demonstrated successfully as a − 0.3 K drop from room temperature in low phonon energy ZBLANP glass doped with Ytterbium ions^2^. The requirement for using low phonon energy materials is linked to the ease with which phonon assisted non-radiative relaxation can counter cooling, unless the bandgap is sufficiently large compared to the phonon energy, making the probability for this detrimental channel to be low^3^. Additionally, any background absorption in the material becomes a parasitic heat load reducing the cooling efficiency significantly^4^. Thus, even high-gain laser-grade materials are not typically of sufficiently high quality for laser cooling applications and care needs to be exercised in purifying materials to reduce impurity-assisted relaxation and background absorption. Until recently, much of the cooling activity was restricted to highly purified crystals with low phonon energies with the preferred dopant Yb. The 4f. electrons responsible for cooling are deeply buried within the electron shell, and thus shielded from the lattice vibrations of the surrounding medium^5^. The ground state to the first excited state energy gap is also the largest of all rare-earth elements, making phonon assisted non-radiative transition less likely and thus a material of choice for laser cooling experiments^6^. While fluoride host materials have been used for the demonstration of cryogenic operation and cooling of thermal loads^7, 8^ many applications motivate the need to broaden the selection of host materials, particularly to include oxide glasses due to their excellent mechanical and optical properties, compatibility with optical fibers and the ease with which they retain their properties through heat treatment, moulding, cutting and polishing. The solubility of rare earths in silica must be increased via the addition of network modifiers^9–11^. The MCVD process has seen an improvement in REE solubility due to the addition of network modifiers in SiO_2_. Al_2_O_3_ and P_2_O_5_ have both been successful individually^12, 13^, as well as when they are combined in specific ratios, forming AlPO_4_ entities^14, 15^. This has resulted in an increase in the silica concentration quenching limit from 10^25^ ions/m^3^ to 10^26–27^ ions/m^3^ ^16, 17^. Although this increases the concentration of rare earths, the high phonon energy environment of the oxide glass also limits the concentration at which quenching occurs^18, 19^. This leads to reduced quantum efficiency through non-radiative interactions and lifetime shortening of the radiative emission from the excited manifold^18^. Thus, high phonon energy and detrimental aggregation of Yb ions encountered at high doping in oxide glasses remain challenging, making it necessary to improve material purification and increase the concentration of the rare earth, principally Yb ions, to improve cooling efficiency.

To counter these limitations, our previous work concentrated on artificially creating a low phonon environment for the rare earth in high phonon energy oxide glasses, by the use of fluoride nanocrystals^20–28^. While this technique showed enhanced quantum efficiency of emission and increased lifetimes, the fabrication route was limited by the purity of starting materials and the process by which the glasses were made^29^, requiring a radical change in the glass making process.

The modified chemical vapour deposition (MCVD) technique using a silica host offers a promising route to intrinsically high purity materials. Alternative routes to enhance the luminescent properties of silica, such as the sol–gel process and integration of nanoparticles, could potentially lead to higher quantum yields and concentrations, as well as greater control over the environment of the rare earth elements^30–33^. Despite showing promise, the results obtained from these methods have not exceeded those produced by standard vapor deposition technique. Recently, laser cooling has been achieved in silica-based glasses by a number of groups^6, 34–36^, with the largest temperature drop reported from room temperature of − 18 K in an alumina rich silica fiber under vacuum using an optimum pump wavelength power of 20 W at 1035 nm^36^. The temperature drop at atmospheric pressure for another fiber was reported to be − 6 K with 185 W of pump power at 1035 nm. These results were obtained by increasing Yb solubility by the addition of alumina and showing the potential for laser cooling in silica glass. The potential for increased dopant concentration for better cooling may be limited due to the phase separation of the rare earth in the silica-rich glass^37^.

In this work, we modify the MCVD approach to achieve the production of silica glass preform with high Yb concentration. We increase the rare earth’s solubility by the addition of alumina but promote increased phase separation of the rare earth in the oxide glass^36, 37^. The fully oxidized active rare-earth ions are immersed in another non-active rare earth to create a local phase separated low phonon energy oxide environment, shielding the rare earth from the detrimental effects of the high phonon energy silica. With this technique, we report a concentration of Yb in the silica host of 2.5 × the highest value reported in the literature^34^ for laser cooling, without significant lifetime shortening or reduction in cooling efficiency. We also target to highlight the effect of ytterbium ion density on the optical properties of GAYY glasses. In addition, all the glasses are doped with 0.15% atomic percent of germanium (Ge) to make it photosensitive for fabricating a self-cooled laser in future. Si is replaced by the Ge (IV) atoms in the glass matrix, and the environment for Yb ions essentially stays the same^37^.

While our preliminary results are with a non-optimized pump wavelength and limited pump power, we show cooling by − 0.9 K using 6.5 W pump power at 1029 nm, which is better than has been reported in the literature to date, to the best of our knowledge. This approach opens the potential for many new applications for rare earth doped oxide glass, not only for cooling but for high-power amplifiers and lasers as well as new wavelength windows for amplification.

## Experimental results and discussion

The investigation of the effect of the network modifier (Al and Y) on the material properties started from the structural characterizations of the rare-earth doped preforms. A series of glasses were prepared in order to study the effect of ytterbium ion concentration on achieving maximum cooling. In addition, a normal silica glass without network modifiers was prepared to study the effect of glass composition on optical properties as well as cooling characteristics. The resulting samples had an 1.5 mm diameter core in the centre and undoped (no Yb doping) silica glass cladding region of 0.3 mm. These were cut and polished into 10 mm long, 2.1 mm square cross-section samples. The fiber preforms along with the ytterbium ion, Al and Y concentrations (in atomic percent), the mean fluorescence wavelength (MFW) and quantum yield at RT while exciting with 1029 nm laser with 5 W pump power are listed in Table 1. The propagation losses of glasses were measured to be 10 ± 3 dB/km.

**Table 1:** The Yb ion concentration of investigated glasses along with the terminology used in the text, Al & Y concentration (in atomic percent), the mean fluorescence wavelength (MFW) & quantum yield at RT while exciting with 1029 nm laser with 5 W pump power.

Yb concentration (ions/m^3^)	Terminology	Al concentration (atomic percent)	Y concentration (atomic percent)	MFW $({\lambda }_{f})$ (nm) (± 0.5 nm)	Quantum yield $({\eta }_{ext})$ at 1029 nm (± 0.5%)
1.3 × 10^26^	GAYY-1	8.5 (± 0.5)%	4.9% (± 0.2)%	1004.60	98.7
2.4 × 10^26^	GAYY-2	8.2 (± 0.4)%	4.5% (± 0.3)%	1006.06	99.1
4.0 × 10^26^	GAYY-3	7.6 (± 0.3)%	4.6% (± 0.2)%	1008.10	99.4
2.4 × 10^26^	MCVD SiO_2_ glass	0	0	1008.60	87.0

**Figure 1:**
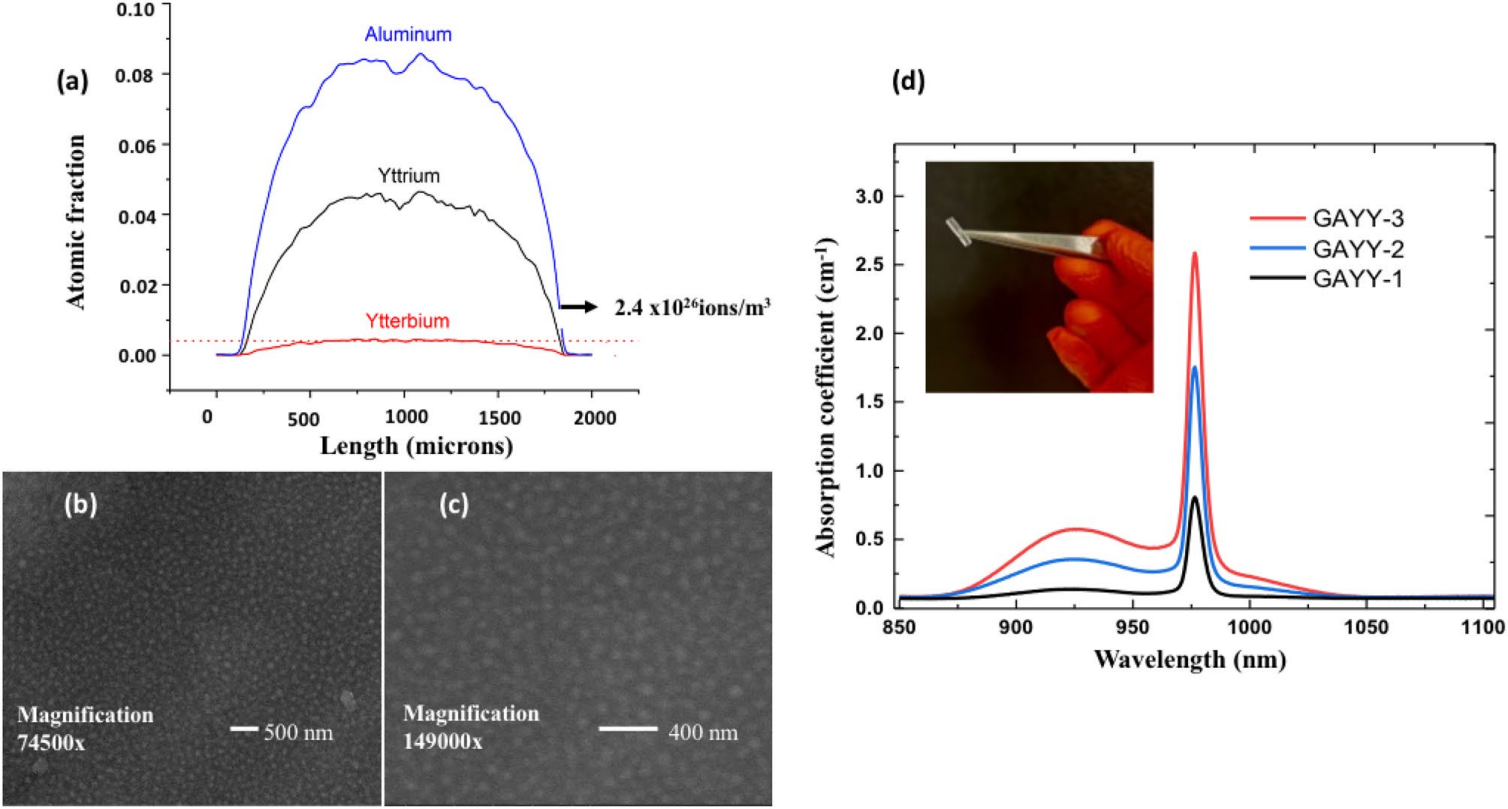
(**a**) EPMA analysis of GAYY-2, (**b**, **c**) SEM Image of phase separated preform (GAYY-2) at different magnifications, using backscattering electron mode (**d**) The absorption spectra of all GAYY glasses. The inset shows the highly transparent GAYY-2 preform.

Electron probe microanalyses (EPMA) were used to assess the average dopant levels within the core region. An example of EPMA is given in Fig. 1a for the GAYY-2 sample. The highest Yb concentration obtained was of 4 × 10^26^ ions/m^3^ at the center of the preform core (GAYY-3) which is the largest value reported in the literature for laser cooling. Figure 1b,c depict the SEM images showing the phase separated Yb-Y-rich regions with an average size of 29 nm. If no external variation (change of tube diameter, precursors with different hydration levels etc.) is applied to process parameters influencing the soot porosity, the final concentration of elements remain stable. We control these parameters using elemental analysis via electron probe microscopy, on slices of preform and refractive index profile. The elemental profiles of the GAYY-2 glass at the bottom and top of the silica tube along with the SEM images are provided in the supplementary information (SI Fig. 1). We observed longitudinal in-homogeneities in every preform, caused by our vertical method of solution doping. The bottom of the tube, where the solution enters usually present 10 to 15% higher concentration of elements, local concentration also present disturbance. On the other side, the top two-third of the tube (from 250 to 600 mm) present higher quality material, with well-dispersed concentrations. For this reason, the samples we use for fiber drawing and bulk preparation are extracted from the top part of the preform, where the homogeneity is better.

A very interesting feature of this particular glass is the phase separation. The majority of commercially available Yb fibres are co-doped with Al because it is an extremely important RE ion solubilizer in silica^10, 36, 37^. While phase separation is usually considered as a detrimental to luminescence, the GAYY glasses exhibit the opposite effect. The excess addition of yttrium creates the phase separation, dissolving ytterbium ions in well-defined regions. The incorporation of alumina plays a promoting role in decreasing the magnitude of the phase separated droplets to tens of nanometers, greatly reducing Rayleigh scattering effects. Transmission electron microscopy (TEM) was used to detect phase separation in fibre preforms at the nanometer scale. Droplet particles enriched in Yb^3+^, Y^3+^, and Al^3+^ were found in the investigated samples in a main silicate phase background. In GAYY glasses, the formation of Yb^3+^-enriched droplets of 29 nm diameter in size was evidenced. As the distance between Yb ions grows, Y and Yb ions can be easily substituted in yttria-alumina silica glass because of their similar ionic radii (104 pm for Y^3+^ and 100.8 pm for Yb^3+^). Similar to the formation of Er–O–Y–O–Er as reported previously^38^, Yb–O–Y–O–Yb forms in such glasses which avoids the daunting challenges associated with the Yb clustering. As a result, the development of oxygen defect centres related to Yb ceases. By mixing fully oxidised active rare-earth ions with another non-active rare earth, low phonon solvation shell around the Yb^3+^ ions are formed which shield rare-earth from the detrimental impact of the high phonon energy silica and increases the luminescence efficiency.

All of the preforms were highly transparent (> 90% in the near infrared region). The Yb^3+^ ion possesses a broad absorption band (Fig. 1d) varying from 850 to 1050 nm, related to the ground (2F_7/2_) and excited (2F_5/2_) Stark sublevel electronic transitions. As the Yb ion concentration in the GAYY glasses increases, the absorption coefficient at 976 nm also increases. The transmittance data of the glasses from VUV to IR region is provided in the supplementary information as SI Fig. 2.

### Spectral properties of GAYY glasses

**Figure 2:**
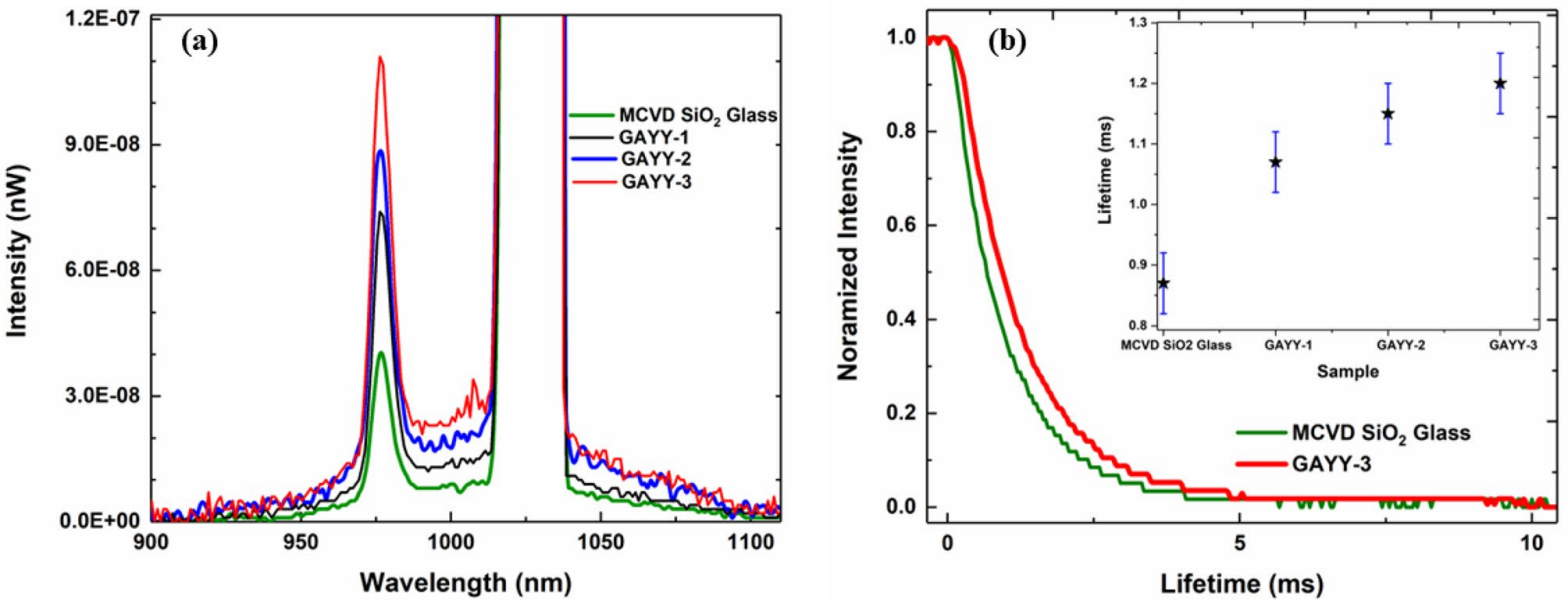
(**a**) The anti-Stokes emission in all the GAYY glasses as well as MCVD SiO_2_ glass while exciting with 1029 nm laser with 5 W pump power, (**b**) The lifetime decay curves of MCVD SiO_2_ glass and GAYY-3 at an excitation wavelength of 920 nm. The inset shows the lifetime values of all glasses.

Excellent optical quality of the GAYY glasses were attested by photoluminescence measurements. The characteristic emission spectra between 900 and 1100 nm for the transition from the 2F_5/2_ manifold to the 2F_7/2_ ground state can be observed in all of the Yb-doped glasses. while pumping with 1029 nm^39^ (Fig. 2a). The PL intensity in GAYY glasses is higher compared to the MCVD SiO_2_ glass under the same illumination and detection conditions. Even if the Yb ion concentration in MCVD SiO_2_ glass and GAYY-2 were almost the same, PL intensity is lower in MCVD SiO_2_ glass due to the concentration quenching effect. This is due to the Yb–O–Yb interaction at high doping levels. In GAYY glasses this interaction is lowered due to the advantage of network modifiers such as Y and Al. Due to the lower phonon energies in GAYY glasses, the rate of multi-phonon relaxations is reduced, resulting in a higher PL emission intensity. The presence of high concentrations of yttrium (III) heavy ions in phase separated droplets leads to a decrease in the maximum phonon energy of silicate oxide glass, which is normally 1100 cm^−1^. As per the Miyakawa and Dexter theory^40^, the rate of multi-phonon decay is exponentially dependent on the difference in energy gap between the energy levels and the maximum phonon energy of the material^41^.

The mean fluorescence wavelength (MFW) of the glass system is determined mostly from the composition and temperature of the host materials. The MFW, ${\lambda }_{f}$ is found by the following equation using the measured fluorescence spectrum while exciting at 1029 nm^6, 42, 43^.

1 $$\lambda_{f} = \frac{{\smallint \lambda I_{f} \left( \lambda \right)d\lambda }}{{\smallint I_{f} \left( \lambda \right)d\lambda }}$$

where ${I}_{f}\left(\lambda \right)$ is the spectral density, measured for our samples by exciting them at 1029 nm. The MFW in GAYY glasses changes from 1004.6 to 1008.1 nm depending on the Yb concentration (Table 1). The optical cooling can occur only when ${\lambda }_{p}$ is greater than ${\lambda }_{f}$. The laser cooling efficiency ${\eta }_{c}$ can be briefly expressed as

2 $$\eta_{c} = \frac{{\lambda_{p} }}{{\lambda_{f} }}p - 1$$

where $p(\lambda )$≲ 1 denotes the probability of converting the absorbed pump photon to an emitting fluorescence photon. According to Eq. (2), the larger the energy difference between the pump and mean fluorescence photons, the higher will be the cooling efficiency. However, due to the reduction in the resonant absorption coefficient at longer wavelengths, increasing the pump wavelength gradually creates a practical limit on pump detuning. Moreover, at higher detuning where resonant absorption is decreased, the effect of the background absorption (contained in the definition of $p\left(\lambda \right)$ begins to dominate, even negating cooling for sufficiently large detuning.

Furthermore, concentration quenching was evident in the quantum yield as well as lifetime measurements in the case of MCVD SiO_2_ glass. The quantum yield of all glasses obtained through integrating sphere method^22,23,28^ while exciting with 1029 nm laser are provided in Table 1. A maximum quantum yield of ~ 99.4% was achieved for GAYY-3 glass. The quantum yield values of the GAYY glasses were higher than that of the MCVD SiO_2_ glass as expected provided by the advantage of low phonon energy environment and high solubility of RE ions which eliminates the concentration quenching effect.

Taking this into consideration, further investigation of the optical properties was achieved for the MCVD SiO_2_ glass and GAYY glasses by examining the lifetime decay curves at an excitation wavelength of 920 nm. Figure 2b shows the lifetime decay curves of MCVD SiO_2_ glass and GAYY-3. The inset in Fig. 2b shows the lifetime of Yb^3+^: 5/2 level in all glasses. The presented ‬curves ‬demonstrate ‬a ‬single ‬exponential ‬decay ‬for Yb^3+^ ions. During the measurement of the intrinsic decay time of the 2F_5/2_ level, care was taken to minimise the impact of various reabsorption processes. In contrast to the GAYY glasses the MCVD SiO_2_ glass without phase separation showed a shortened luminescence decay time of 0.87 ms due to concentration quenching^44^. Since the Yb^3+^ are in a low phonon energy pool of yttrium rich domains the lifetimes of all GAYY glasses are higher than that of the MCVD SiO_2_ glass. Also, the lifetime was observed to be increase from 1.04 to 1.20 ms with the increment in ytterbium ion concentration provided by the prominent advantage of networking modifiers (Al and Y)^45^. This enhancement in lifetime would also highlight the lower probability of non-radiative energy transfer and therefore higher quantum efficiency. The lifetime is expected to be decreased in the quenching regime^44, 46^.

‬Another finding that further supports the above description is the presence of higher cooperative emission intensity in the MCVD SiO_2_ glass compared to the GAYY glasses. Cooperative luminescence at a wavelength of 500 nm was used to probe Yb^3+^ clustering in the MCVD SiO_2_ glass. It is a viable technique to indicate the formation of Yb^3+^ pairs and clusters in glasses. When two Yb^3+^ ions are simultaneously de-excited, cooperative luminescence occurs, which causes the emission of one photon with a shorter wavelength in the visible spectrum. Figure 3 in supplementary information shows the emission spectra of the MCVD SiO_2_ glass and GAYY-3 with 400 mW of absorbed pump power at 980 nm wavelength. The cooperative emission intensity decreases in GAYY-3 due to the higher RE ion solubility ascribed to the presence of Al and Y.

**Figure 3:**
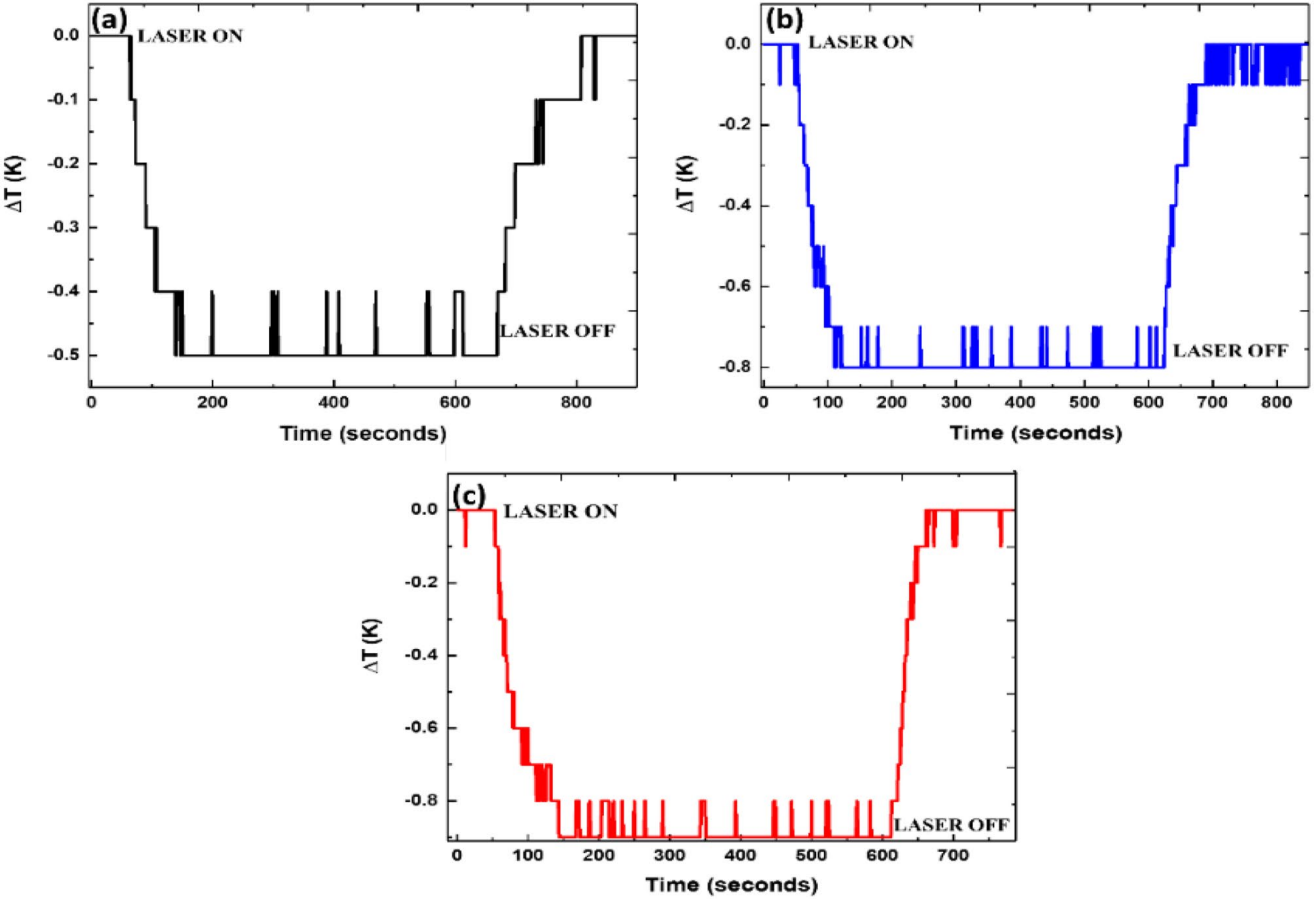
The temperature dynamics of (**a**) GAYY-1, (**b**) GAYY-2, and (**c**) GAYY-3 and while pumping with 6.5 W 1029 nm laser.

The enhanced lifetime and quantum yield in GAYY glasses is expected to enable their use in a wide range of optical devices and to provide significant insights in the design of glassy materials for radiation balanced lasers and fibre amplifiers. Apart from that, the GAYY glasses possessed higher purity which make it highly promising for laser cooling applications. The use of MCVD process allows us to achieve higher purity glasses which is essential to observe cooling. Since one of the primary sources of loss that contributes to heating is background absorption, worldwide efforts have been concentrated on different purification techniques to be used to fabricate laser cooling materials^47, 48^. Hopefully, the characteristics of MCVD make it possible to reduce the absorption of impurities in our GAYY glasses. The background absorption coefficient of 10 dB/km determined using calorimetric technique^49, 50^ shows that the investigated glasses are highly promising for cooling. We deepened the insights into background absorption using elemental analysis done through by inductively coupled plasma (ICP) measurements. The amount of impurities such as iron or rare earths measured by ICP mass spectrometry showed less than 200 ppb of both Fe/Cu (major impurities which are detrimental for cooling)^51^.

### Laser induced cooling in GAYY glasses

To investigate the potential of fabricated glasses for laser cooling we performed the temperature measurements on all glasses at an excitation wavelength of 1029 nm with different pump power varying from 0.5 W to 6.5 W. A 1029 nm laser was the only high-power pump source (6.5 W) that was accessible in our lab. Since this pump wavelength is higher than that of the mean fluorescence wavelengths (≲ 1008.6 nm), a net heat extraction was obtained in all GAYY glasses. The temperature dynamics of all the GAYY glasses while exciting with a 6.5 W 1029 nm laser is shown in Fig. 3. The pump laser is kept off for ~ 1 min, and then it is turned on for ~ 10 min. The GAYY glass with ytterbium ion concentration of 4.0 × 10^26^ ions/m^3^ showed a − 0.1 K cooling using 0.5 W of pump power and reached to a maximum of − 0.9 K from RT using 6.5 W at atmospheric pressure. A maximum temperature reduction of − 0.5 and − 0.8 K from RT was achieved in the case of GAYY-1 and GAYY-2 glasses respectively. Since only the core of the preform is doped with ytterbium the heat extraction only tends to happen there, however the entire glass cools evenly in 1 min (Fig. 3).

The thermal coupling time constant of the GAYY-3 was estimated to be *τ*_*t*_ = 31 s. The overall heat transfer coefficient *h*_*eff*_ was approximated as ${h}_{eff}={h}_{cv}+4{\sigma }_{B}\varepsilon {T}_{r}^{3}$ in which *h*_*cv*_ is the convective heat transfer coefficient, $\epsilon$ is the hemispherical emissivity of the sample, ${\sigma }_{B}$ is the Stefan–Boltzmann constant and *T*_*r*_ is the ambient room temperature. According to lumped-capacitance model^41, 49^ the ${h}_{eff}=C/({\tau }_{t}{A}_{surf})$= 23.39 Wm^−2^ K^−1^, where *C* is the heat capacity of the preform. Using the model described in Ref. 31 & 39 the heat load ${P}_{load}=-{A}_{surf}{h}_{eff}\Delta T$ was found to be 1.4 mW where *A*_*surf*_ is the surface area of the preform and *∆T* = *T*_*r*_*–T*_*s*_. The thermal parameters of the GAYY-3 glass is summarized in Table 2.

**Table 2:** The thermal parameters of GAYY-3 glass.

Parameter	Value	Units
Mass density *ρ*	2750	kg m^−3^
Specific heat *c*_*h*_	680	J kg^−1^ K^−1^
Heat capacity *C*	0.049	J K^−1^
Surface area *A*_*surf*_	7.3 × 10^−5^	m^2^
Thermal coupling time constant ${\tau }_{h}$	31	s

The steady state temperature measured using FBG contact technique^49, 50^ was further supported by the FLIR thermal camera images. The resolution of the temperature change measured using the FBG method as well as using the FLIR is 0.1 K. The thermal camera images of the GAYY-3 while exposed to 1029 nm laser is shown in supplementary information Fig. 4. The thermal camera image darkens after being exposed to the 1029 nm wavelength laser which can be easily observed by the unaided human eye (see around the crosshair in each picture). There is a 0.2 K discrepancy between the measurements using FBG and thermal camera due to the poor resolution as well as lesser emissivity options available in the thermal camera^41^. However, they are widely utilized in optical cooling experiments due to the ease of handling^2, 6^.

**Figure 4:**
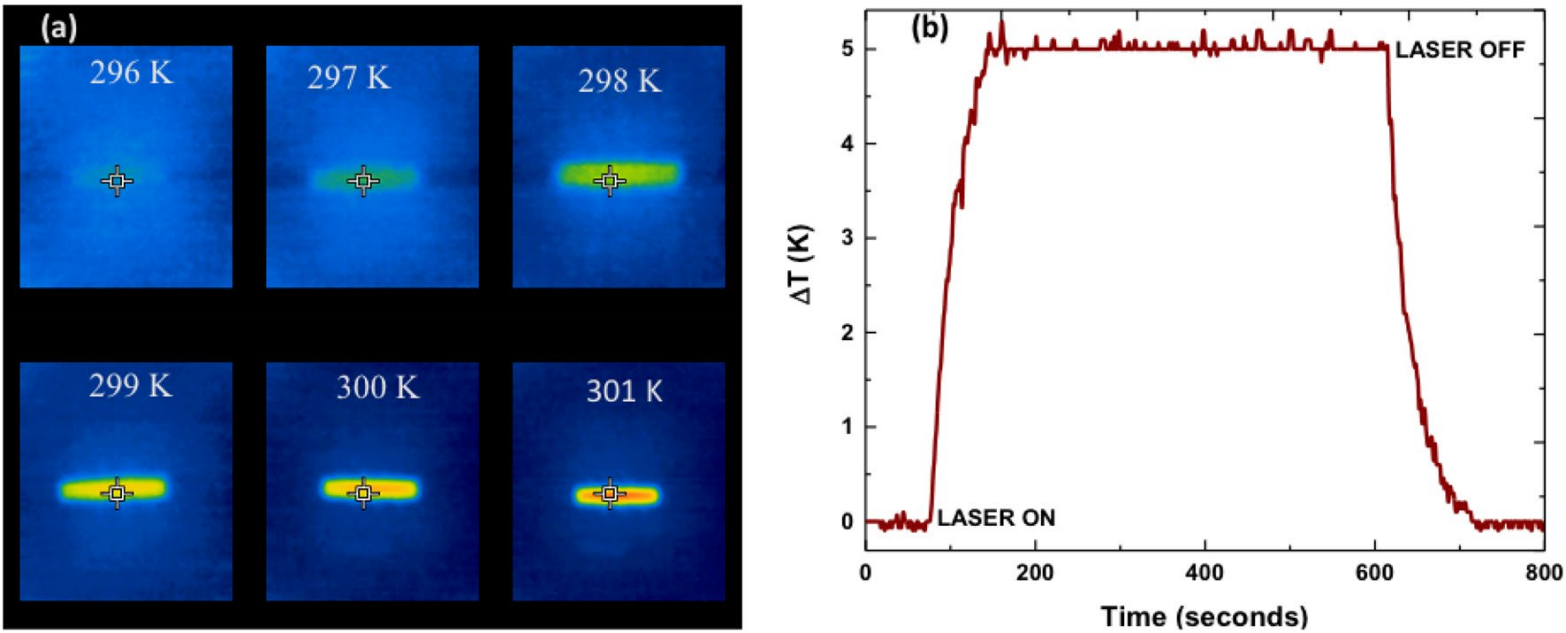
(**a**) The temperature evolution of MCVD SiO_2_ glass while it is exposed to 1029 nm 1 W laser obtained through FLIR thermal camera, (**b**) $\Delta \mathrm{T}$ vs. time curve of MCVD SiO_2_ glass while pumping with 1 W at 1029 nm measured using FBG direct contact technique.

We performed a comparative study between the glasses having different cladding diameter in order to evaluate the effect of heat load using GAYY-2 glass. During the initial laser cooling experiments, we used samples that were surrounded by an undoped silica glass cladding region of 0.5 mm, which was a significant thermal load. Despite this substantial thermal loading, a cooling of − 0.5 K from RT was achieved with a pump power of 6.5W. Later, in order to lower the thermal load and enhance cooling, we removed the majority of its undoped cladding region, and it showed a − 0.8 K temperature difference from RT using the same pump conditions. On the contrary, the MCVD SiO_2_ glass sample heated to ~ 5 K using a pump power of only 1 W. This is the most remarkable outcome of our study, namely, that the GAYY glass demonstrated such a dramatic difference compared to the normal preform while pumping with 1029 nm laser. The heating is associated with the concentration quenching effect. The thermal camera images of the MCVD SiO_2_ glass while exposed to 1029 nm laser is shown in Fig. 4a. The temperature rises to 301 K from 296 K. The temperature dynamics is depicted in Fig. 4b. Here the amount of absorbed pump power before the Yb absorption reaches saturation is greatly limited. In this case, the additional pump power is absorbed mainly by the background impurities and efficiently converts pump power to heat^52^. Therefore, even if the purity of the MCVD SiO_2_ glass without phase separation is high, cooling would be hindered due to quenching.

### Theoretical model and numerical results

The full potential of these findings was determined by a simulation, since we were unable to increase the pump power being limited by our available laser. The cooling efficiency, defined in Eq. (2) can be expanded as follows

3 $$\eta_{c} = {\raise0.7ex\hbox{${P_{cool} }$} \!\mathord{\left/ {\vphantom {{P_{cool} } {P_{abs} }}}\right.\kern-0pt} \!\lower0.7ex\hbox{${P_{abs} }$}} = \frac{{\lambda_{p} }}{{\lambda_{f} }}\eta_{abs} \eta_{ext} - 1,$$

where the absorption efficiency can be expressed as

4 $$\eta_{abs} = \frac{{\alpha_{r} }}{{\alpha_{r} + \alpha_{b} }}.$$

Also ${\alpha }_{r}$ is the resonant absorption coefficient and ${\alpha }_{b}$ is the background absorption coefficient. The quantum efficiency can be approximated as^28, 53–55^

5 $$\eta_{ext} \cong \frac{{\gamma_{rad} }}{{\gamma_{rad} + \gamma_{q} }} = \left[ {1 + \frac{9}{2\pi }\left( {\frac{N}{{N_{c} }}} \right)^{2} } \right]^{ - 1} ,$$

where ${\gamma }_{rad}$ and ${\gamma }_{q}$ are radiative and quenching rate respectively. *N* is the density of ions and *N*_*c*_ is the critical density of ions due to the quenching. Rewriting Eq. (5), the critical concentration, *N*_*c*_ is as follows

6 $$N_{c} = N\sqrt {\frac{{\frac{9}{2\pi } \eta_{ext} }}{{\left( {1 - \eta_{ext} } \right)}}} .$$

We calculated the critical concentration corresponding to the concentration of 4.0 × 10^26^ ions/m^3^ for GAAY-3 and found it to be 6.16 × 10^27^ ions/m^3^ which is ~ 5 times larger than the highest value reported in literature (i.e., 1.2 × 10^27^ ions/m^3^)^34^.

Next, we determine the temperature drop for each pump power at 1029 nm excitation using a numerical calculation. Refs.^56,57^ introduced and established a two-level model for absorption and emission processes between the Yb^3+^ 2F_7/2_ ground-state manifold and the 2F_5/2_ excited-state manifold. The cooling power of the preform can be calculated using the following equation^58^

7 $$P_{cool} = \frac{{a_{eff } \sigma_{a} \left( {T_{s} } \right) N I_{s} \left( {T_{s} } \right) \left( {\lambda /\lambda_{f*} \left( {T_{s} } \right) - 1} \right)}}{{1 + \sigma_{e} \left( {T_{s} } \right)/\sigma_{a} \left( {T_{s} } \right) + a_{eff } I_{s} \left( {T_{s} } \right)/P}}$$

where ${I}_{s}({T}_{s})=hc{\gamma }_{rad}/{\lambda }_{p}{\sigma }_{a}({T}_{s})$ is the saturation intensity, ${\lambda }_{f*}({T}_{s})$ is the effective mean fluorescence wavelength, and ${\sigma }_{a}({T}_{s})$ and ${\sigma }_{e}({T}_{s})$ are the absorption and the emission cross sections, respectively. *P* is the pump power and ${a}_{eff}$ is the effective pump mode area. The sum of radiative and convective heat load on the sample is given by

8 $$P_{load} = A_{surf} \epsilon \sigma_{B} \left( {T_{r}^{4} - T_{s}^{4} } \right) + A_{surf} h_{cv} \left( {T_{r} - T_{s} } \right)$$

**Figure 5:**
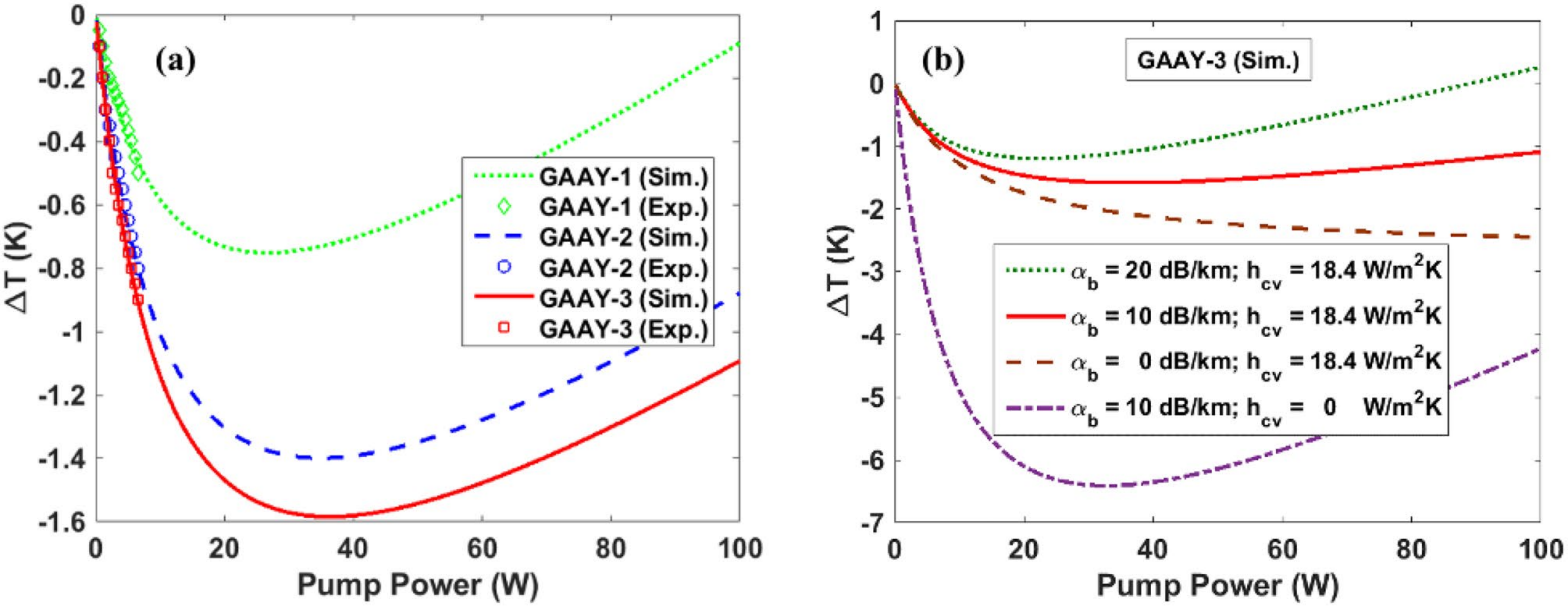
(**a**)The temperature change as a function of the pump power in GAYY glasses in air. The markers show the experimental results for the pump powers up to 6.5 W, (**b**) The effect of background absorption and convective heat load in obtaining temperature reduction with a change in pump power in GAYY-3 glass.

At thermal equilibrium*, P*_*cool*_ = *P*_*load*_ allows the determination of temperature drop for each pump power for a given pump wavelength using a numerical calculation. Figure 5a shows temperature change curves as a function of the pump power in GAYY glasses in air considering ${h}_{cv}$ = 18.4 W/m^2^ K and the background absorption coefficient of 10 dB/km. Maximum temperature drops of − 0.75, − 1.40 and − 1.58 K are expected at 26.6, 35.1 and 36.1 W for GAYY-1, GAYY-2 and GAYY-3, respectively. Further increase in the pump power leads to heating in the sample due the pump absorption by background impurities. The experimentally obtained values illustrated by markers on each curves, are in excellent agreement with the simulation.

For practical applications it is essential to demonstrate ASF cooling in atmospheric pressure since it can be useful to make self-cooled lasers and amplifiers. For our sample having a convective heat transfer coefficient of 18.4 W/m^2^ K, the maximum obtainable temperature reduction is also limited by the background absorption. For a given pump power, higher purity results in more temperature reduction as depicted in Fig. 5b. Due to the convective heat load while performing optical cooling experiments at atmospheric pressure, most of the experiments are performed in vacuum^3, 4, 6, 36^. Figure 5b shows that a maximum temperature reduction of − 6.4 K for a 32.6 W pump power is predicted for GAYY-3 if we conduct the experiments in vacuum, i.e., *h*_*cv*_ = 0. A − 3.95 K drop in temperature in vacuum for a 6.5 W pump power is also predicted. However, at atmospheric pressure, i.e., *h*_*cv*_ = 18.4 w/m^2^ K, for the GAYY-3 having ${\alpha }_{b}=10\mathrm{ dB}/\mathrm{km}$, the expected temperature drop is ~ 1.58 K for a pump power of 32.6 W. The temperature drop is even smaller i.e., 1.13 K, if ${\alpha }_{b}$ is 20 dB/km. It is clear that the background absorption plays a critical role in achieving maximum temperature reduction.

We have thus realized optical cooling in highly Yb doped silica glasses that will find broad applications in solid-state laser cooling, radiation balanced lasers, amplifiers etc. GAYY glass is unique from other Yb doped silica glasses explored in the past for laser cooling applications due to its higher quantum efficiency and longer lifetime due to it’s controlled phase separation effect which hold Yb^3+^ ions in yttrium-rich domains, compared to regular silica glass. Because of the increased Y_2_O_3_ concentration, these areas showed not only significantly higher rare-earth solubility but also much smaller phonon energy in comparison with a pure silica matrix. We have demonstrated the ability to regulate the local environment of Yb^3+^ ions by dissolving them in domains containing a high concentration of yttrium, which benefit from the immiscibility between rare earth elements and silica. By adding Al_2_O_3_, the scale of this phase separation was reduced to nanometer-sized particles, resulting in significantly diminishing Rayleigh diffusion effects in the Near Infra-Red region. This approach is distinctly different to the more traditional approach, in which phase separation is considered a nuisance and is avoided.

Further optimization efforts will focus on the use of several excitation wavelengths longer than that of 1029 nm, increasing the pump power, optimizing the sample geometry etc. We have fabricated optical fibers out of the GAYY glasses and temperature measurements are in progress. Longer term research may additionally involve the exploration of optical fibres made out of GAYY glasses for radiation balanced fiber lasers. Since the cooling GAYY glass preform is doped with Ge, the glass is photosensitive enough to make reflective gratings in order to fabricate RBLs. Also, the proposed approach for achieving high emission efficiency will be extended to other rare earth materials with different network modifiers to achieve cooling.

## Conclusions

In this work, we show laser cooling in Yb-doped 100% oxide glasses with different ytterbium concentration. A maximum temperature reduction of − 0.9 K from room temperature at atmospheric pressure was obtained experimentally using only 6.5 W of 1029 nm pump radiation. A maximum temperature reduction of − 6.4 K for a 32.6 W pump power is predicted in vacuum. The temperature change versus pump power was also presented using a numerical simulation that well agrees with the experimental ones. The results also demonstrated that, despite its high phonon energy, silica can tolerate unexpectedly high Yb concentrations without compromising its optical properties as demonstrated by cooling a glass with 4 × 10^26^ ions/m^3^. We have reported cooling in the highest concentration of Yb ions (a ~ 2.5 × improvement) with little or no degradation in lifetime or quantum efficiency, compared to the best results previously reported in the literature. This incredible achievement was made possible by tailoring the material to substantially lower non-radiative relaxation sources, such as concentration quenching and background absorptive loss. Co-doping the glass with Al and Y, resulted in a record-breaking critical concentration, *N*_*c*_ that is ~ 5 times higher than the previous reports. Combining the optical absorption, photoluminescence, and fluorescence decay data, the effect of ytterbium ion concentration on the spectroscopic properties and cooling properties of glasses were studied systematically. The cooperative emission intensity decreases in GAYY glass compared to MCVD SiO_2_ glass which indicates a reduction of Yb^3+^ pairs due to the presence of network modifiers. Our findings indicate that this novel glass is a highly suitable candidate for radiation-balanced lasers and amplifiers. It also opens up some intriguing possibilities for further research into more glass compositions with various RE ions and network modifiers suitable for laser cooling applications.

### Materials and characterization methods

The glasses used in this work were fabricated using modified chemical vapour deposition (MCVD) technique^59, 60^. The detailed procedure is provided in supplementary information (SI5).

The microstructural analysis of the preform was performed using scanning electron microscopy (SEM) with an FEI Quanta 3D FEG, using 10 kV voltage excitation, backscattering electrons. To prevent charge accumulation on the sample, an 8 nm thick Pt/Rh layer was applied to the polished preform cores. The elemental analysis was carried out using electron probe microscopy on a Castaing CAMECA-SX100. Absorption coefficient spectra were obtained from transmission measurements on slices of preforms. Parallel slices of preform ranging from 1.5 to 2 mm in thickness are taken and optically polished. Samples are then processed using a Carry 5000 UV–Vis spectrometer for the 200–1500 nm range, and on a Perkin Elmer FTIR spectrometer from 1500 to 5000 nm. Also, the photoluminescence (PL) emission spectra as well as the quantum yield of the samples were measured using a 1029 nm laser (Pharos). A Thorlabs IS200-4 integrating sphere was connected to an Ando AQ6317B optical spectrum analyzer (OSA) via a multimode optical fiber in order to gather the emitted light^22, 23^.

Fluorescence excitation and time-decay characteristics associated to the Yb^3+^: 2F_5/2_ level were measured using a 920 nm pump from a Ti: Sapphire laser. The luminescence lifetime ($\tau )$ values were acquired using a fast oscilloscope (Tektronix). The signal was recorded using a photodiode (Thorlabs SM05PD1B) and then amplified by a benchtop trans-impedance amplifier. The elimination of the pump wavelength was achieved by using an edge filter, while the modulation of the external signal was done by applying a Thorlabs MC100A chopper with two slot blades operating at a frequency of 20.0 Hz^27^. The background absorptive loss measurements were made using fiber Bragg grating (FBG) direct contact technique with an IPG ELR-70-1550-LP 1550 nm ytterbium-erbium fiber laser at 5 W, as a source of optical power^49^.

**Figure 6:**
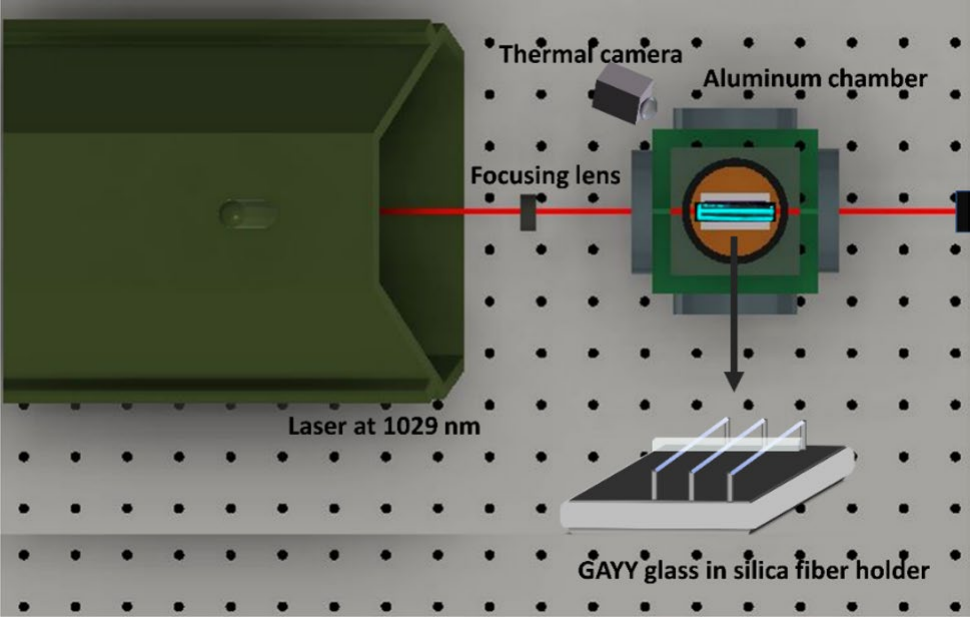
The experimental setup used for temperature measurements in laser cooling experiment. The zoomed-in circle shows GAYY glass placed on the silica optical fibres.

Laser cooling measurements were done by evaluating the steady-state temperature change of the glasses while exciting with a 1029 nm laser (Pharos) with varying pump powers ranging from 0.5 to 6.5 W. The maximum peak power density of 1.708e + 8 W/cm^2^ used in our measurements showed no optical damage. The samples were supported by a pair of silica optical fibers, which were adhered to a stainless-steel holder located inside an aluminum chamber to minimize the heat load on the samples. A schematic of the experiment is depicted in Fig. 6. To monitor the temperature, the fiber Bragg grating (FBG) direct contact method^27, 49, 50^ was employed. The shift in central wavelength of the FBG is directly proportional to the temperature change of the sample. A thermal camera (FLIR) was also employed to measure the temperature change upon laser irradiation at 1029 nm.

## Supplementary Information

Supplementary Information.

## Data Availability

Data underlying the results presented in this paper are not publicly available at this time but may be obtained from the corresponding author upon reasonable request.
