# Supplementary material for: Demonstration of laser cooling in a novel all oxide GAYY silica glass
Source: Sci Rep. 2023 Apr 3;13:5436. doi: 10.1038/s41598-023-31912-1 (PMC10070423; doi:10.1038/s41598-023-31912-1)
Supplement: Supplementary file 1 — Supplementary Information. [file 41598_2023_31912_MOESM1_ESM.docx]

**Supplementary Information**


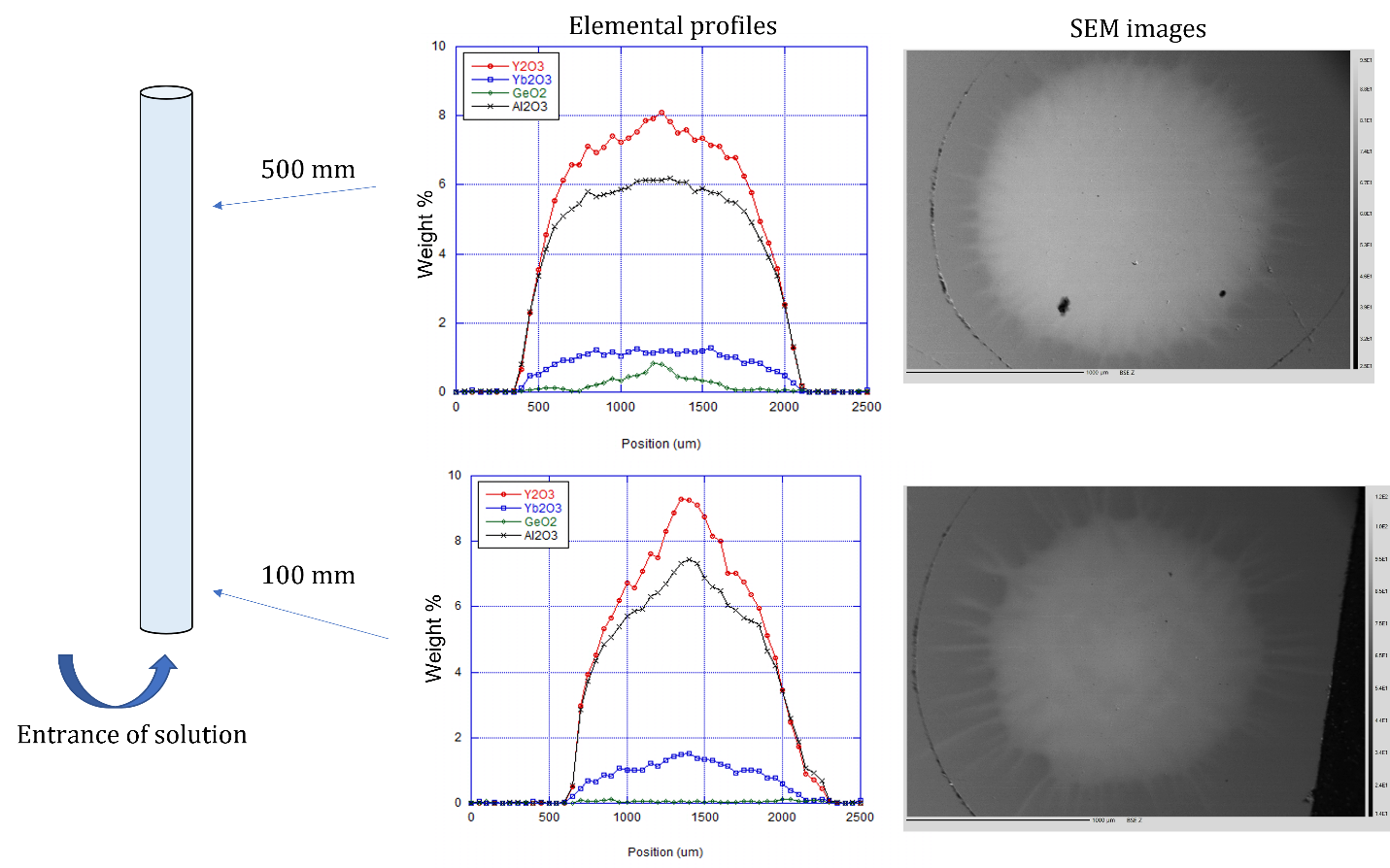


**SI Figure 1**: The elemental profiles of the GAYY-2 glass at the bottom and top of the silica tube along with the SEM images.





**SI Figure 2:** The transmission of the glasses from VUV to IR region





**SI Figure 3**: Cooperative luminescence spectra of MCVD SiO_2_ glass and GAYY-3 with 400 mW of absorbed pump power while exciting with 980 nm laser.


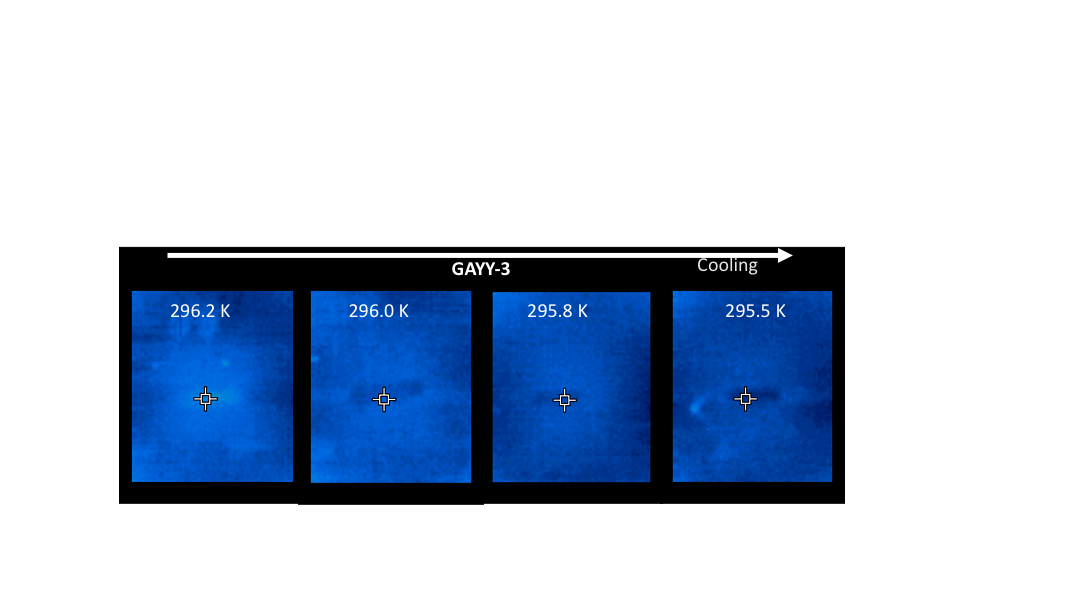


**SI Figure 4**: The temperature evolution of GAYY 3 glass while it is exposed to 1029 nm 6.5 W laser obtained through FLIR thermal camera.

**SI 5: Glass fabrication using modified chemical vapor deposition (MCVD) process**

A F300 grade Heraeus silica tube was placed inside of an MCVD system. After cleaning and drying steps, SiCl_4_ and He/O_2_ gas flowed through the tube. The tube was locally heated to 1325°C with a H_2_/O_2_ burner to oxidize the silicon tetrachloride into a SiO_2_ soot. The flame is moved along the tube to create a first layer of SiO_2_ on the inner walls of the tube. This step is then repeated two more times. The doping elements were added by an aqueous solution of 6N chloride salts of Al, Y and Yb (99.9999% Pure Analytical Laboratories). The solution flowed through the porous silica soot and left for 20 minutes. The solution is then removed and a 2L/min flow of dry N2 passed for 12h. The silica tube is then re-placed on the MCVD bench and dried at high temperature with multiple passes from 800 to 1500°C. Sintering and vitrification of the soot is realized at 1600°C, followed by a 2100°C pass. Collapse of the preform is performed from 2100 to 2200°C. The final preform is then fire polished at 2000°C.
